# Results from a multicenter, noninterventional registry study for multiple myeloma patients who received stem cell mobilization regimens with and without plerixafor

**DOI:** 10.1038/s41409-019-0676-0

**Published:** 2019-09-18

**Authors:** Curly Morris, Christian Chabannon, Tamas Masszi, Nigel Russell, Hareth Nahi, Guido Kobbe, Marta Krejci, Holger W. Auner, David Pohlreich, Patrick Hayden, Grzegorz W. Basak, Stig Lenhoff, Nicolaas Schaap, Anja van Biezen, Cora Knol, Simona Iacobelli, Qianying Liu, Marina Celanovic, Laurent Garderet, Nicolaus Kröger

**Affiliations:** 1 Queens University, Belfast, UK; 2 Institut Paoli-Calmettes, Marseille, France; 3 Semmelweis University, Budapest, Hungary; 4 Nottingham University, Nottingham, UK; 5 Karolinska University Hospital, Stockholm, Sweden; 6 University Hospital of Dusseldorf, Dusseldorf, Germany; 7 Department of Internal Medicine, Hematology and Oncology, University Hospital Brno and Faculty of Medicine, Masaryk University, Brno, Czech Republic; 8 Imperial College London, London, UK; 9 Charles University Hospital, Prague, Czech Republic; 10 St. James Hospital, Dublin, Ireland; 11 Medical University of Warsaw, Warsaw, Poland; 12 Skanes University Hospital, Lund, Sweden; 13 Radboud University Medical Centre, Nijmegen, The Netherlands; 14 European Society for Blood and Marrow Transplantation, Leiden, The Netherlands; 15 University Tor Vergata, Rome, Italy; 16 Sanofi, Cambridge, MA USA; 17 Hospital Saint Antoine, Paris, France; 18 University Hospital, Hamburg, Germany

**Keywords:** Medical research, Phase IV trials

## Abstract

Plerixafor plus granulocyte-colony stimulating factor (G-CSF) enhances the mobilization of hematopoietic stem cells (HSCs) for collection and subsequent autologous hematopoietic stem cell transplantation (HSCT) in patients with multiple myeloma (MM). This international, multicenter, noninterventional registry study (NCT01362972), evaluated long-term outcomes for MM patients who received plerixafor versus other mobilization regimens. The comparisons were: G-CSF + plerixafor (G-CSF + P) versus G-CSF-; G-CSF + P versus G-CSF + chemotherapy (G-CSF + C); and G-CSF + P + C versus G-CSF + C. Propensity score matching was used to balance groups. Primary outcome measures were progression free survival (PFS), overall survival (OS), and cumulative incidence of relapse (CIR) after transplantation. After propensity matching, 77 versus 41 patients in the G-CSF + P versus G-CSF cohorts, 129 versus 129 in the G-CSF + P versus G-CSF + C cohorts, and 117 versus 117 in the G-CSF + P + C versus G-CSF + C cohorts were matched, respectively. Propensity score matching resulted in a smaller sample size and imbalances were not completely overcome. For both PFS and OS, the upper limits of the hazard ratio 95% confidence intervals exceeded prespecified boundaries; noninferiority was not demonstrated. CIR rates were higher in the plerixafor cohorts. G-CSF + P remains an option for the mobilization of HSCs in poor mobilizers with MM with no substantial differences in PFS, OS, and CIR in comparison with other regimens.

## Introduction

Plerixafor (Mozobil®) in combination with granulocyte-colony stimulating factor (G-CSF) is approved by the European Medicines Agency to enhance the mobilization of hematopoietic stem cells (HSCs) to the peripheral blood for collection and subsequent autologous transplantation in patients with lymphoma and multiple myeloma (MM) who are recognized as poor mobilizers of HSCs. Plerixafor is a selective, reversible inhibitor of the chemokine receptor 4 with a (C-X-C) motif (CXCR4) and has a unique mechanism of action compared with other HSC mobilizing agents [1, 2]. The C-X-C motif chemokine 12/CXCR4 receptor interaction is an integral part of the retention of HSCs in the bone marrow and inhibition of this interaction by plerixafor temporarily mobilizes HSCs from the bone marrow to the peripheral blood [3, 4].

There is a theoretical risk of tumor cell mobilization with any stem cell mobilization method for hematopoietic stem cell transplantation (HSCT). Therefore, the European Union mandated analysis of the European Society for Blood and Marrow Transplantation (EBMT) data registry to evaluate the long-term outcomes of patients with MM who had received plerixafor.

The long-term clinical outcomes collected in this postapproval analysis of the EBMT registry included an evaluation of progression free survival (PFS), overall survival (OS), and the cumulative incidence of relapse (CIR) in patients with MM who had undergone mobilization, collection, and transplantation of autologous blood progenitors. The analysis evaluated patients who received plerixafor for stem cell mobilization and HSCT and compared their outcomes with those of patients who had received other mobilization regimens.

## Methods

### Study design

This was an international, multicenter, noninterventional registry study with patient follow-up of 3.5–7.5 years to evaluate the long-term outcomes of MM patients who received plerixafor for stem cell mobilization and who completed their first autologous HSCT between 2008 and 2012 (ClinicalTrials.gov number NCT01362972). The analysis included a prospectively defined cohort of MM patients with data reported retrospectively to the EBMT. Patients from Austria, Belgium, Bulgaria, Czech Republic, Finland, France, Germany, Greece, Hungary, Ireland, Israel, Italy, Netherlands, Poland, Romania, Spain, Sweden, Switzerland, and the United Kingdom were included in the study. Eligibility included all patients’ ≥18 years of age from the EBMT registry with a diagnosis of MM who were to receive an autologous HSCT and were transplanted. This was a noninferiority study. The noninferiority margin was assigned as a 30% increase in PFS and OS corresponding to a hazard ratio (HR) upper limit of 1.3. No lower limit was set. Summary curves for CIR were planned. Due to the observational nature of the study, no formal statistical hypothesis testing was planned with adequate power and the type I error control.

The study was conducted in accordance with the Declaration of Helsinki and the International Conference on Harmonization Guidelines for Good Clinical Practice. For all sites, approval of the protocol was obtained from the governmental authorities and Institutional Review Boards.

### Poor mobilizers

Predicted poor mobilizers were patients who had received prior irradiation to marrow-bearing areas or who had high exposure to marrow-damaging chemotherapy. Proven poor mobilizers were patients who, in previous mobilization attempts, had failed to mobilize sufficient CD34+ cells to the peripheral blood to proceed to apheresis or transplantation, or who, in the current mobilization, had failed to achieve a sufficient rise in peripheral blood CD34+ at the predicted time of peak mobilization.

### Data collection

The data were entered, managed, and maintained in a central database with internet access. Data were retrieved from variables identified on the EBMT Medical A and Medical B forms and Medical C form for poor mobilization data.

### Outcomes

The primary efficacy outcomes were OS, PFS, and CIR. Key secondary efficacy outcomes were hematological recovery (time to absolute neutrophil counts of ≥0.5 × 10^6^/l and platelet reconstitution of ≥50 × 10^9^/l). The key safety outcome was transplant complications occurring from the day of transplantation until 100 days post transplant.

The following mobilization regimens were compared:

1. G-CSF plus plerixafor (G-CSF + P) compared with G-CSF alone.
2. G-CSF + P compared with G-CSF plus chemotherapy (G-CSF + C).
3. G-CSF plus plerixafor plus chemotherapy (G-CSF + P + C) compared with G-CSF + C.

Graft failure was defined as no engraftment (neutrophils never reached ≥0.5 × 10^9^ cells/l) or graft loss (neutrophils reached ≥0.5 × 10^9^ cells/l but subsequently decreased to a lower level of cells until additional engraftment treatment was given).

### Statistical analyses

In nonrandomized clinical studies, differences in baseline characteristics between treatment groups may influence outcomes, leading to bias [5]. The propensity score, defined as the individual probability of receiving a treatment based on the baseline characteristics of the patient, is intended to reduce bias when assessing outcomes between two treatments [5].

Propensity score method was used to identify study comparison groups that were balanced with respect to baseline characteristics, including, demographics, MM disease type, disease characteristics and staging, bone marrow involvement, prior treatment characteristics, and disease status [5]. The baseline variables and patient demographics used for propensity score matching are shown in Table 1. Only patients who were identified as a “Proven or Predicted Poor Mobilizer” were included in the analysis.

**Table 1:** Patient demographics used for propensity score matching in the matched comparison groups

	Comparison 1		Comparison 2		Comparison 3	
	G-CSF + plerixafor (*N* = 77)	G-CSF alone (*N* = 41)	G-CSF + plerixafor (*N* = 129)	G-CSF + chemo (*N* = 129)	G-CSF + plerixafor + chemo (*N* = 117)	G-CSF + chemo (*N* = 117)
Age at diagnosis, mean years ± standard deviation	57.5 ± 8.4	56.1 ± 8.1	58.2 ± 7.8	58.6 ± 7.0	60.0 ± 7.5	59.6 ± 7.1
Age at first mobilization, mean years ± standard deviation	58.2 ± 8.4	57.0 ± 8.4	59.3 ± 7.8	59.5 ± 6.8	61.3 ± 6.9	60.6 ± 6.8
Female sex, *n* (%)	37 (48.1)	21 (51.2)	43 (33.3)	42 (32.6)	55 (47.0)	59 (50.4)
Bone marrow involvement at the start of mobilization, *n*/*N* (%)	9/55 (16.4)	4/31 (12.9)	32/95 (33.7)	37/108 (34.3)	43/93 (46.2)	35/99 (35.4)
Status of disease at collection, *n* (%)	*N* = 60	*N* = 37	*N* = 102	*N* = 100	*N* = 87	*N* = 89
Complete response or stringent complete response	8 (13.3)	6 (16.2)	13 (12.7)	10 (10.0)	5 (5.7)	6 (6.7)
Partial response	44 (73.3)	26 (70.3)	73 (71.6)	80 (80.0)	77 (88.5)	81 (91.0)
Stable disease	6 (10.0)	4 (10.8)	8 (7.8)	7 (7.0)	3 (3.4)	1 (1.1)
Progressive disease	2 (3.3)	1 (2.7)	8 (7.8)	3 (3.0)	2 (2.3)	1 (1.1)
Durie and Salmon disease stage at diagnosis, *n* (%)	*N* = 68	*N* = 40	*N* = 115	*N* = 115	*N* = 91	*N* = 105
A-1	2 (2.9)	3 (7.5)	8 (7.0)	8 (7.0)	11 (12.1)	8 (7.6)
A-II	8 (11.8)	7 (17.5)	15 (13.0)	24 (20.9)	17 (18.7)	24 (22.9)
A-III	43 (63.2)	23 (57.5)	68 (59.1)	64 (55.7)	53 (58.2)	63 (60.0)
B-I	0.0	0.0	2 (1.7)	0.0	0.0	0.0
B-II	3 (4.4)	0.0	5 (4.3)	1 (0.9)	0.0	0.0
B-III	12 (17.6)	7 (17.5)	17 (14.8)	18 (15.7)	10 (11.0)	10 (9.5)
Interval between diagnosis and first collection, mean months ± SD	9.2 ± 10.8	10.7 ± 10.6	13.0 ± 19.8	10.0 ± 21.0	14.1 ± 36.5	11.1 ± 22.3
Multiple graft programme, *n* (%)						
Yes	1 (1.3)	1 (2.6)	1 (0.8)	4 (3.1)	8 (6.8)	13 (11.1)
No	75 (98.7)	38 (97.4)	127 (99.2)	124 (96.9)	109 (93.2)	104 (88.9)
Year of first transplant						
2008	8 (10.4)	13 (31.7)	8 (6.2)	8 (6.2)	5 (4.3)	9 (7.7)
2009	20 (26.0)	8 (19.5)	32 (24.8)	35 (27.1)	13 (11.1)	17 (14.5)
2010	20 (26.0)	11 (26.8)	24 (18.6)	28 (21.7)	31 (26.5)	40 (34.2)
2011	18 (23.4)	6 (14.6)	32 (24.8)	33 (25.6)	30 (25.6)	29 (24.8)
2012	11 (14.3)	3 (7.3)	33 (25.6)	25 (19.4)	38 (32.5)	22 (18.8)
Geographic region						
South and East Europe	18 (23.4)	11 (26.8)	32 (24.8)	30 (23.3)	34 (29.1)	37 (31.6)
North and West Europe	59 (76.6)	30 (73.2)	97 (75.2)	99 (76.7)	83 (70.9)	80 (68.4)
Multiple myeloma disease types, *n* (%)						
IgG	42 (54.5)	22 (53.7)	78 (60.5)	81 (62.8)	63 (53.8)	65 (55.6)
IgA	12 (15.6)	8 (19.5)	16 (12.4)	17 (13.2)	32 (27.4)	30 (25.6)
Light chain (kappa and lambda)	20 (26.0)	9 (22.0)	32 (24.8)	28 (21.7)	15 (12.8)	17 (14.5)
IgD, IgE, or IgM	2 (2.6)	1 (2.4)	2 (1.6)	3 (2.3)	6 (5.1)	3 (2.6)
Nonsecretory	1 (1.3)	1 (2.4)	1 (0.8)	0.0	1 (0.9)	2 (1.7)

A single imputation approach was implemented to create complete data sets for analyses. Propensity scores were then fitted using logistic regression models. Matches for plerixafor patients were identified from the nonplerixafor groups based on the estimated propensity scores. Matching was performed without replacement. Model success was based on whether balance between the plerixafor and the control groups matched samples was achieved. In the original design, the plan was to have two nonplerixafor patients identified for each plerixafor patient. However, it was not possible as there were many more plerixafor patients than nonplerixafor patients in patients who were predicted or proven poor mobilizers. In particular, in the group of patients who did not receive chemotherapy, matching was performed for two plerixafor patients with one nonplerixafor patient.

Following the propensity score analysis, the outcomes for each mobilization treatment group were analyzed for comparable groups. Cox proportional hazards model with covariates was used for OS and PFS. The 95% confidence intervals (CI) and HR for the effect of treatment were calculated. Survival curves were developed for each treatment group using nonparametric Kaplan–Meier estimates [6]. A competing risk model was developed for CIR; death without prior progression/relapse was treated as a competing event. The 95% CI and cumulative incidence at each year post transplantation were estimated.

Due to the observational nature of the study, sample size was not calculated based on power calculations. It has been estimated using the following assumptions: that 85% of transplanted MM patients would receive G-CSF + C and 15% would receive G-CSF alone; that 10% of transplanted patients with each regimen would be treated with plerixafor; and that 70% of plerixafor patients would be matched at a ratio of 1:2 plerixafor to comparator. It was estimated that 4600 patients would be included in the study over a 5-year period and would include: 100 patients in the G-CSF alone group, 540 patients in the G-CSF + C group, 50 patients in the G-CSF + P group, and 270 patients in the G-CSF + P + C group. The predicted number of events for the outcome analysis was 101 for the G-CSF + P compared with G-CSF alone, 101 for the G-CSF + P compared with G-CSF + C, and 546 for the G-CSF + P + C compared with the G-CSF + C group.

## Results

### Participants and demographics

**Fig. 1:**
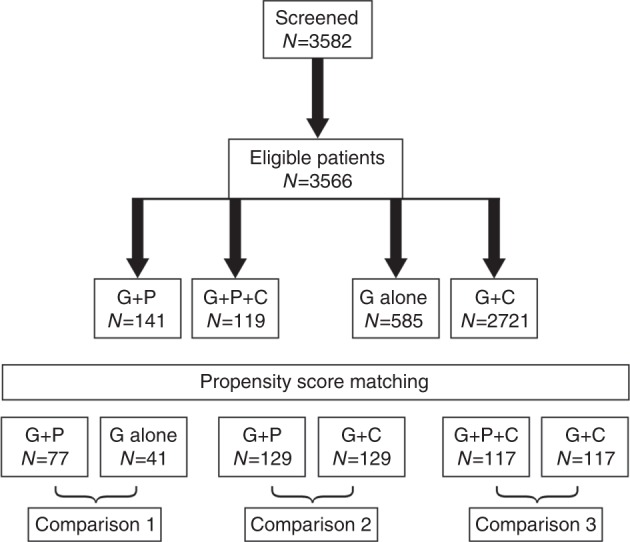
Patient eligibility and treatment

Overall, 3582 MM patients were screened and, of these, 3566 patients met the study eligibility criteria. These included 141 patients treated with G-CSF + P, 119 patients treated with G-CSF + P + C, 585 patients treated with G-CSF alone, and 2721 patients treated with G-CSF + C (Fig. 1).

Baseline demographic and disease history data used in propensity scoring are summarized in Table 1. The groups were well matched on age and sex and were comparable for Durie and Salmon disease staging, with the majority of patients in each group assessed as Stage III (IIIA or IIIB). At mobilization, the proportion of patients with bone marrow involvement ranged from 12.9 to 46.2% across the groups (Table 1).

The propensity scoring of poor mobilizers identified matched groups for the comparative analysis (Table 2). After propensity scoring, 77 versus 41 patients were matched in the G-CSF + P versus G-CSF alone cohort, 129 versus 129 in the G-CSF + P versus G-CSF + C cohort, and 117 versus 117 in the G-CSF + P + C versus G-CSF + C cohort. The three groups treated with plerixafor had greater proportions of patients, prior to administration of plerixafor, who failed to mobilize sufficient CD34+ cells at the predicted peak mobilization time compared with the comparison groups (Table 2).

**Table 2:** Mobilization characteristics for the matched comparison groups

	Comparison 1		Comparison 2		Comparison 3	
	G-CSF + plerixafor (*N* = 77)	G-CSF alone (*N* = 41)	G-CSF + plerixafor (*N* = 129)	G-CSF + chemo (*N* = 129)	G-CSF + plerixafor + chemo (*N* = 117)	G-CSF + chemo (*N* = 117)
Myelosuppressive chemotherapy prior to first mobilization, *n*/*N* (%)	74/75 (98.7)	29/29 (100)	123/124 (99.2)	108/109 (99.1)	105/106 (99.1)	105/106 (99.1)
Predicted poor mobilizers^a^, *n*/*N* (%)	13/71 (18.3)	9/35 (25.7)	23/122 (18.9)	47/126 (37.3)	18/116 (15.5)	47/114 (41.2)
Proven poor mobilizers^b^, *n*/*N* (%)	77/77 (100.0)	35/41 (85.4)	128/128 (100)	93/129 (72.1)	115/117 (98.3)	85/117 (72.6)
Prior medical history, *n*/*N* (%)						
Failed to mobilize sufficient CD34+ cells to proceed to apheresis	36/63 (57.1)	17/30 (56.7)	52/101 (51.5)	60/80 (75.0)	41/79 (51.9)	56/69 (81.2)
Failed to collect sufficient cells to proceed to transplant	14/46 (30.4)	22/27 (81.5)	24/78 (30.8)	38/74 (51.4)	22/71 (31.0)	38/71 (53.5)
Current mobilization during the study						
Failed to mobilize sufficient CD34+ cells at predicted time, *n*/*N* (%)	34/56 (60.7)	7/30 (23.3)	61/93 (65.6)	7/70 (10.0)	56/81 (69.1)	6/61 (9.8)
CD34+ cell count, median (×10^9^/l)	8.0 (*n* = 43)	27.5 (*n* = 20)	9.0 (*n* = 75)	27.0 (*n* = 27)	6.3 (*n* = 60)	25.1 (*n* = 28)
Interval between diagnosis and first transplant, months mean ± SD	12.1 ± 13.9	12.8 ± 10.9	15.6 ± 21.2	12.6 ± 21.4	16.1 ± 36.9	14.0 ± 22.6

^a^Predicted poor mobilizers were defined by the clinicians as patients who had received prior irradiation to marrow-bearing areas or had high exposure to marrow-damaging chemotherapy

^b^Proven poor mobilizers were defined as patients who, in a previous mobilization attempt, failed to mobilize sufficient CD34+ cells in the peripheral blood to proceed to apheresis or to proceed to transplantation or who, in the current mobilization, failed to achieve a sufficient rise in peripheral blood CD34+ at the predicted time for peak mobilization

### Primary endpoints

#### Progression free survival

**Fig. 2:**
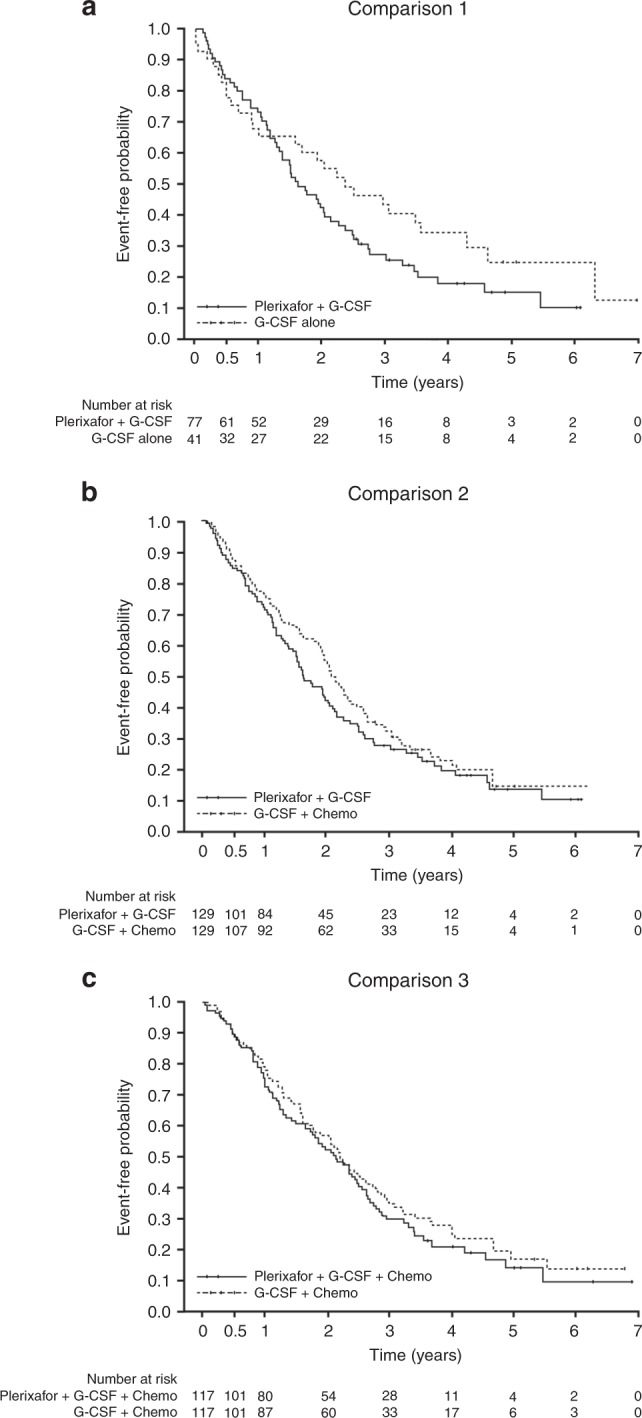
Progression (event) free survival for each of the comparison groups, G-CSF plus plerixafor versus G-CSF alone (comparison 1); G-CSF plus plerixafor versus G-CSF plus chemotherapy (comparison 2); G-CSF plus plerixafor plus chemotherapy versus G-CSF plus chemotherapy (comparison 3)

The estimated 3-year PFS for the G-CSF + P group was 0.27 [95% CI: 0.17, 0.38] versus 0.43 [0.27, 0.58] for G-CSF alone (comparison 1, Table 3); G-CSF + P group was 0.27 [95% CI: 0.19, 0.36] versus 0.32 [95% CI: 0.24, 0.41] for the G-CSF + C group (comparison 2), and G-CSF + P + C group was 0.29 [95% CI:0.21, 0.38] versus 0.34 [95% CI: 0.25, 0.43] for the G-CSF + C group (comparison 3). Due to the small sample size, the 95% confidence limits of the HRs for PFS and OS were wide and the upper limits of the 95% CI were >1.3, based on prespecified boundaries; the plerixafor-containing groups did not therefore fulfill the criteria for noninferiority compared with the comparator groups. Kaplan–Meier survival curves showed that PFS in the plerixafor groups was generally lower (Fig. 2).

**Table 3:** Primary outcomes: progression free survival, overall survival and cumulative incidence of relapse for each of the comparator groups

	Comparison 1		Comparison 2		Comparison 3	
	G-CSF + plerixafor (*N* = 77)	G-CSF alone (*N* = 41)	G-CSF + plerixafor (*N* = 129)	G-CSF + chemo (*N* = 129)	G-CSF + Plerixafor + chemo (*N* = 117)	G-CSF + chemo (*N* = 117)
Estimated PFS, 3 years [95% CI]	0.27 [0.17, 0.38]	0.43 [0.27, 0.58]	0.27 [0.19, 0.36]	0.32 [0.24, 0.41]	0.29 [0.21, 0.38]	0.34 [0.25, 0.43]
Estimated 3-year overall survival [95% CI]	0.67 [0.55, 0.77]	0.67 [0.50, 0.79]	0.72 [0.62, 0.79]	0.79 [0.71, 0.85]	0.73 [0.64, 0.81]	0.80 [0.71, 0.86]
Deaths, *n* (%)	36 (46.8)	15 (36.6)	53 (41.1)	42 (32.6)	38 (32.5)	37 (31.6)
Hazard ratio [95% CI]	1.44 [0.78, 2.66]		1.13 [0.72, 1.76]		1.25 [0.79, 1.98]	
Cumulative incidence of relapse, 3 years, [95% CI]	0.72 [0.59, 0.81]	0.50 [0.33, 0.64]	0.71 [0.61, 0.79]	0.65 [0.56, 0.74]	0.69 [0.59, 0.77]	0.61 [0.51, 0.70]

#### Overall survival

**Fig. 3:**
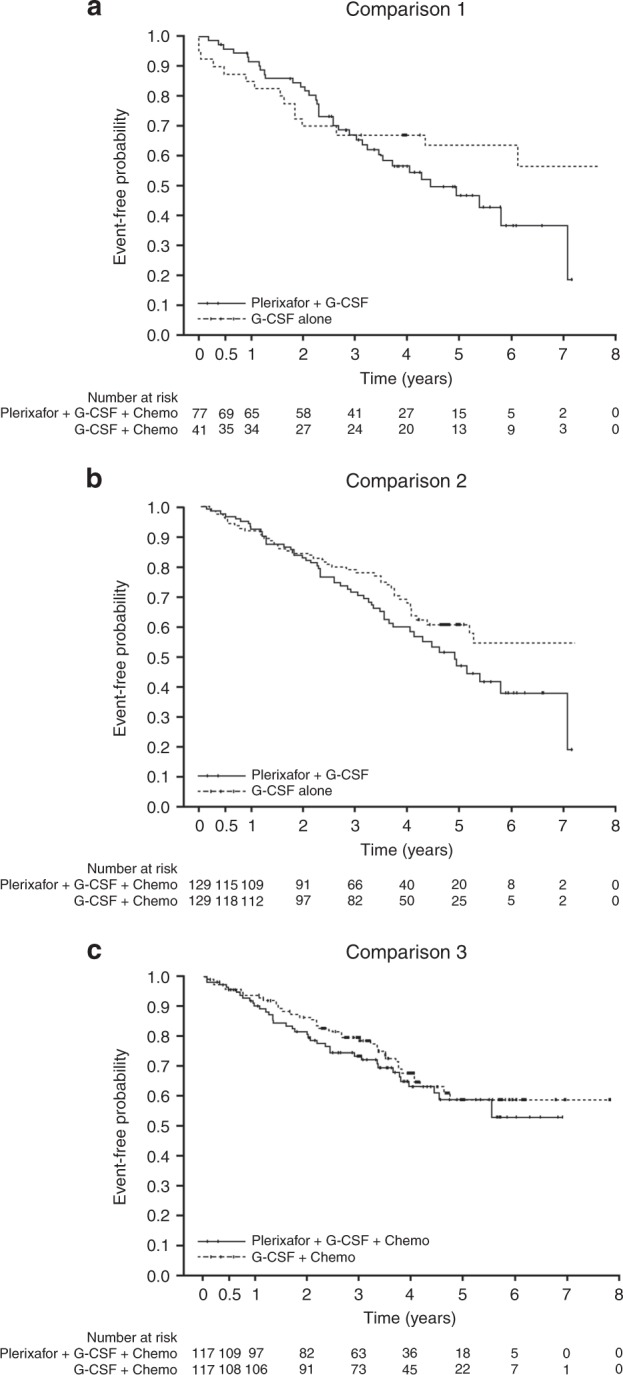
Overall (event-free) survival for each of the comparison groups, G-CSF plus plerixafor versus G-CSF alone (comparison 1); G-CSF plus plerixafor versus G-CSF plus chemotherapy (comparison 2); G-CSF plus plerixafor plus chemotherapy versus G-CSF plus chemotherapy (comparison 3)

The results for OS are shown in Table 3. Kaplan–Meier survival curves showed that OS in the plerixafor groups was generally lower as time progressed (Fig. 3). As the upper limit of the HR was >1.3, based on predetermined boundaries, noninferiority of plerixafor was not demonstrated for any of the comparison groups.

#### Cumulative incidence of relapse

**Fig. 4:**
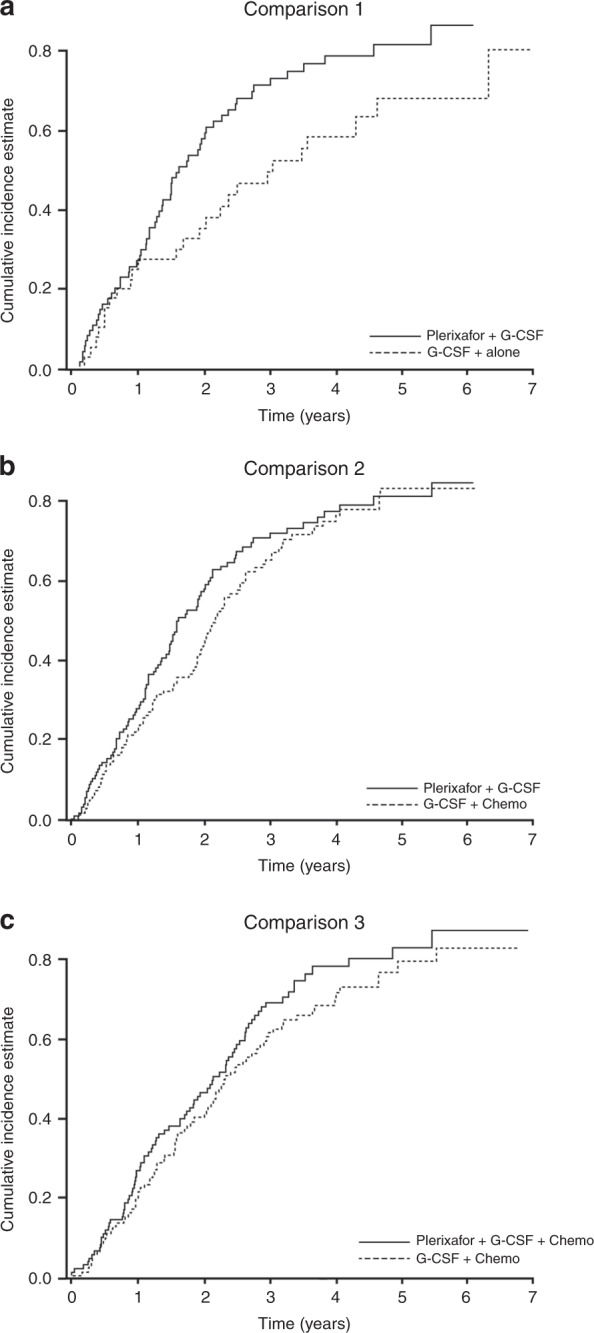
Cumulative incidence of relapse with death without progression/relapse as a competing risk for each of the comparison groups, G-CSF plus plerixafor versus G-CSF alone (comparison 1); G-CSF plus plerixafor versus G-CSF plus chemotherapy (comparison 2); G-CSF plus plerixafor plus chemotherapy versus G-CSF plus chemotherapy (comparison 3)

A competing risk model was used to determine the CIR, which appeared slightly higher in the plerixafor groups compared with the comparator group (Fig. 4).

### Secondary outcomes

#### Post transplantation

Adverse events occurring in more than one patient in any treatment group up to 100 days post first transplantation are shown in Table 4. Infections and infestations were the most common standard organ class complication in all plerixafor and comparator groups (Table 4).

**Table 4:** Adverse events occurring in more than one patient in any treatment group up to 100 days post first transplantation

	Comparison 1		Comparison 2		Comparison 3	
Adverse event, *n* (%) Standard organ class, *n* (%)	G-CSF + plerixafor (*N* = 77)	G-CSF alone (*N* = 41)	G-CSF + plerixafor (*N* = 129)	G-CSF + chemo (*N* = 129)	G-CSF + plerixafor + chemo (*N* = 117)	G-CSF + chemo (*N* = 117)
Any adverse event up to 100 days post transplantation	34 (44.2)	20 (48.8)	61 (47.3)	55 (42.6)	49 (41.9)	50 (50.4)
Infections and infestations	30 (39.0)	18 (43.9)	51 (39.5)	43 (33.3)	38 (32.5)	48 (41.0)
Blood and lymphatic disorders	2 (2.6)	0	4 (3.1)	6 (4.7)	3 (2.6)	7 (6.0)
Gastrointestinal disorders	2 (2.6)	0	4 (3.1)	1 (0.8)	3 (2.6)	3 (2.6)
General disorders and administration site conditions	10 (13.0)	5 (12.2)	17 (13.2)	15 (11.6)	13 (11.1)	14 (12.0)
Metabolism and nutritional disorders	N/A	N/A	1 (0.8)	3 (2.3)	0	3 (2.6)
Renal and urinary disorders	0	1 (2.4)	2 (1.6)	0	0	1 (0.9)
Nervous system disorders	1 (1.3)	2 (4.9)	1 (0.8)	1 (0.8)	1 (0.9)	1 (0.9)
Cardiac disorders	0	1 (2.4)	1 (0.8)	2 (1.6)	1 (0.9)	2 (1.7)
Vascular disorders	0	2 (4.9)	1 (0.8)	3 (2.3)	3 (2.6)	2 (1.7)

Engraftment was reported for ≥95% of patients in the groups in each of the three paired comparisons. Collectively, the median number of days to achieve a neutrophil count of ≥0.5 × 10^9^/l was 12 days, and a platelet count of ≥20 × 10^9^/l was 13 days.

## Discussion

This was a postapproval study in the European Union to monitor for recurrence or progression of myeloma as a surrogate marker of tumor cell contamination of autologous peripheral blood stem cell harvests when using plerixafor in stem cell mobilization regimens. Due to the observational nature and the small sample size, no firm conclusions could be drawn from this study, although the cohorts treated with plerixafor had a trend towards shorter PFS and OS times and a higher CIR, safety outcomes were similar to their respective comparators.

In line with the licensed therapeutic indication for the use of plerixafor in patients with MM, all patients in the primary analysis had to be poor mobilizers, either as predicted poor mobilizers through exposure to high-dose chemotherapy, or proven poor mobilizers based on their mobilization history. Despite propensity scoring, there were more proven poor mobilizers in the plerixafor cohorts, which may have influenced the outcomes due to an imbalance between comparison groups. A further difference between the plerixafor and comparison cohorts was that the median CD34+ cell counts in the plerixafor group during the current study mobilization were lower compared with comparison cohorts. These differences suggested that the groups may not have been balanced for disease prognosis, which may be important, as it has been reported that in poor mobilizers (defined as patients with a collection yield of <4 × 10^6^ CD34+ cells/kg), the time to disease progression, PFS, and OS are all significantly shorter compared with successful mobilizers [7]. In our study, the shorter PFS, OS, and higher CIR for those who received plerixafor compared with the comparison cohorts may, in part, be due to poorer mobilizers in the plerixafor groups.

Even with the introduction of novel agents, including proteasome inhibitors and immunomodulatory drugs, into first-line MM therapy, autologous transplantation remains a cornerstone of treatment for transplant eligible patients [8]. Estimates of the proportion of patients failing to mobilize adequate numbers of stem cells for successful transplantation vary considerably [9–11]. Furthermore, it is now recognized that a second autologous HSCT has a role to play in the management of patients having a response to a first autologous HSCT of >1 year [12–14]. Second HSCT is now recommended for some patients by the National Institute for Clinical Health and Excellence [15] and the International Myeloma Working Group [13] guidelines, and it is probable that a substantial proportion of plerixafor mobilized patients will have obtained sufficient stem cells to facilitate this approach.

The possible effect of the infusion of tumor cells on long-term outcomes should be interpreted with caution, as unfavorable outcomes may simply reflect more aggressive disease as well as factors inherent to tumor cell mobilization. It is important to note that it is very likely that some of the patients in our study would probably not have proceeded to autologous transplantation without plerixafor treatment [16–18]. In support of our findings, results from a 5-year, long-term, phase III, follow-up study (not restricted to poor mobilizers) showed that the use of G-CSF + P did not have a negative effect on PFS and OS in patients with MM, with more than half of all patients with MM still alive 5 years following transplantation [19]. Furthermore, a major concern in the mobilization of stem cells for transplantation is the potential risk of tumor cell mobilization. In this respect, a recent study reported that MM tumor cells were not detected in the apheresis products of patients who received either G-CSF + P or those who received G-CSF alone [20]. Collectively, the findings from these two studies further support the safety of G-CSF + P for mobilizing CD34+ cells for transplantation in poor mobilizers. It is therefore more likely that relapse occurs due to clonal evolution of tumor cells within the patient at the time of transplantation.

Propensity scoring is increasingly used in nonrandomized clinical trials to assess small treatment effects that may introduce potential bias and imbalances between treatment cohorts due to an imbalance of baseline covariates, such as disease state and the number of previous failed mobilizations [5]. In this respect, propensity scoring matched patients for disease and response status, but was unable to match the risk level of the patients, because the genetic risk evaluation based on fluorescent in situ hybridization cytogenetics was not reported, and this may have impacted on the results of our study. Genetic risk evaluation is an important omission, because it is now well established that genetic aberrations are a major prognostic factor for disease progression in patients with MM [21–23]. Patients with high-risk genetic abnormalities benefit less from HSCT, and there is a substantial impact on the survival of these patients [21, 22]. Specifically, point mutations that affected transcriptional regulation of genes have been shown to negatively impact on event-free survival and OS in MM patients [21, 22]. Therefore, it is important to ensure that treatment groups are genetically balanced for those at low risk and at high risk of disease progression. Also, propensity scores cannot adjust for unreported differences between groups and, therefore, this is both a limitation and a source of potential bias in the use of propensity scores [24].

In our study, propensity scoring led to modest size comparisons but maintained an adequate balance for certain key disease characteristics collected in the database. The cohort with the worse results (G-CSF + P versus G-CSF alone) was the group with the smallest sample size—a factor that introduced a large variability. The sample size resulting from propensity score matching could be one of the limitations for the demonstration of noninferiority in the current study. Some important variables, such as the amount of prior chemotherapy, cytogenetic risk, or the use of consolidation and maintenance treatments, could not be incorporated in the model due to the lack, or limited availability, of the data. This imbalance in the data collection could have had a substantial impact on outcomes in our study.

There are a number of other limitations to our study that mandate caution when interpreting the results. The proportion of patients in the primary analysis who were proven poor mobilizers was numerically greater in the plerixafor cohorts (98.3–100%) compared with the comparator cohorts (72.1–85.4%). There were higher numbers of patients in the plerixafor cohorts who failed to mobilize sufficient CD34+ cells at the predicted peak mobilization times. In line with reimbursement of medicine costs in many European countries, clinicians may have selectively given plerixafor to patients who were the poorest mobilizers at the highest risk of poor outcomes, and this may have contributed to the trend for worse outcomes in the plerixafor cohorts [25–29]. A further consideration is that propensity score matching led to modest numbers of patients in the comparison groups, which may have had an effect on the outcomes of the study. There may have been selection bias, as only patients with successful mobilization were included in the study. The observational nature of the study may also have introduced bias, although every effort was made, potential biases may not be removed completely in observational data.

## Conclusions

The findings from this study should be interpreted with caution due to its observational nature, the small sample size of the comparison groups, and the wide 95% CI observed in the HRs. The absence of information on genetic risk and maintenance treatment are further limitations of the study. The cohorts treated with plerixafor had a trend toward numerically shorter PFS and OS times and higher CIR, with similar safety outcomes compared with their respective comparators. Poor mobilization is associated with more aggressive disease and hence poor mobilizers are potentially predisposed to worse outcomes, as may be indicated by the lower baseline CD34+ cell counts at predicted peak of mobilization in the plerixafor cohort [30]. G-CSF + P remains an additional option for the mobilization of HSCs in poor mobilizers with MM with no substantial differences in PFS, OS, and CIR in comparison with other regimens.

## Data Availability

Qualified researchers may request access to patient level data and related study documents including the clinical study report, study protocol with any amendments, blank case report form, statistical analysis plan, and dataset specifications. Patient level data will be anonymized and study documents will be redacted to protect the privacy of trial participants. Further details on Sanofi’s data sharing criteria, eligible studies, and process for requesting access can be found at: https://www.clinicalstudydatarequest.com.

## References

[1] DiPersio JF, Uy GL, Yasothan U, Kirkpatrick P (2009). Plerixafor. Nat Rev Drug Discov.

[2] Nervi B, Link DC, DiPersio JF (2006). Cytokines and hematopoietic stem cell mobilization. J Cell Biochem.

[3] Fruehauf S, Seeger T (2005). New strategies for mobilization of hematopoietic stem cells. Future Oncol.

[4] Lapidot T, Dar A, Kollet O (2005). How do stem cells find their way home?. Blood.

[5] Pirracchio R, Resche-Rigon M, Chevret S (2012). Evaluation of the propensity score methods for estimating marginal odds ratios in case of small sample size. BMC Med Res Methodol.

[6] Kaplan EL, Meier P (1958). Nonparametric estimation from incomplete observation. J Am Stat Assoc.

[7] Brioli A, Perrone G, Patriarca F, Pezzi A, Nobile F, Ballerini F (2015). Successful mobilization of PBSCs predicts favorable outcomes in multiple myeloma patients treated with novel agents and autologous transplantation. Bone Marrow Transpl.

[8] Shah N, Callander N, Ganguly S, Gul Z, Hamadani M, Costa L (2015). Hematopoietic stem cell transplantation for multiple myeloma: guidelines from the American Society for Blood and Marrow Transplantation. Biol Blood Marrow Transpl.

[9] Lee KH, Jung SK, Kim SJ, Jang JH, Kim K, Kim WS (2014). Incidence and risk factors of poor mobilization in adult autologous peripheral blood stem cell transplantation: a single-centre experience. Vox Sang.

[10] Sancho JM, Morgades M, Grifols JR, Junca J, Guardia R, Vives S (2012). Predictive factors for poor peripheral blood stem cell mobilization and peak CD34(+) cell count to guide pre-emptive or immediate rescue mobilization. Cytotherapy.

[11] Wuchter P, Ran D, Bruckner T, Schmitt T, Witzens-Harig M, Neben K (2010). Poor mobilization of hematopoietic stem cells-definitions, incidence, risk factors, and impact on outcome of autologous transplantation. Biol Blood Marrow Transpl.

[12] Cook G, Williams C, Brown JM, Cairns DA, Cavenagh J, Snowden JA (2014). High-dose chemotherapy plus autologous stem-cell transplantation as consolidation therapy in patients with relapsed multiple myeloma after previous autologous stem-cell transplantation (NCRI Myeloma X Relapse [Intensive trial]): a randomised, open-label, phase 3 trial. Lancet Oncol.

[13] Giralt S, Garderet L, Durie B, Cook G, Gahrton G, Bruno B (2015). American Society of Blood and Marrow Transplantation, European Society of Blood and Marrow Transplantation, Blood and Marrow Transplant Clinical Trials Network, and International Myeloma Working Group Consensus Conference on salvage hematopoietic cell transplantation in patients with relapsed multiple myeloma. Biol Blood Marrow Transpl.

[14] Maybury B, Cook G, Pratt G, Yong K, Ramasamy K (2016). Augmenting autologous stem cell transplantation to improve outcomes in myeloma. Biol Blood Marrow Transpl.

[15] Pratt G, Morris TC (2017). Review of the NICE guidelines for multiple myeloma. Int J Lab Hematol.

[16] Kim JS, Yoon DH, Park S, Yoon SS, Cho SG, Min CK (2016). Prognostic factors for re-mobilization using plerixafor and granulocyte colony-stimulating factor (G-CSF) in patients with malignant lymphoma or multiple myeloma previously failing mobilization with G-CSF with or without chemotherapy: the Korean multicenter retrospective study. Ann Hematol.

[17] Sheppard D, Bredeson C, Huebsch L, Allan D, Tay J (2014). A plerixafor-based strategy allows adequate hematopoietic stem cell collection in poor mobilizers: results from the Canadian Special Access Program. Bone Marrow Transpl.

[18] Spoerl S, Peter R, Wascher D, Gotze K, Verbeek M, Peschel C (2017). Patients’ outcome after rescue plerixafor administration for autologous stem cell mobilization: a single-center retrospective analysis. Transfusion.

[19] Micallef IN, Stiff PJ, Nademanee AP, Maziarz RT, Horwitz ME, Stadtmauer EA (2018). Plerixafor plus granulocyte colony-stimulating factor for patients with non-hodgkin lymphoma and multiple myeloma: long-term follow-up report. Biol Blood Marrow Transpl.

[20] Nahi H, Celanovic M, Liu Q, Lund J, Peceliunas V (2019). A pilot, exploratory, randomized, phase ii safety study evaluating tumor cell mobilization and apheresis product contamination in patients treated with granulocyte colony-stimulating factor alone or plus plerixafor. Biol Blood Marrow Transpl.

[21] Chang H, Qi XY, Samiee S, Yi QL, Chen C, Trudel S (2005). Genetic risk identifies multiple myeloma patients who do not benefit from autologous stem cell transplantation. Bone Marrow Transpl.

[22] Avet-Loiseau H, Attal M, Moreau P, Charbonnel C, Garban F, Hulin C (2007). Genetic abnormalities and survival in multiple myeloma: the experience of the Intergroupe Francophone du Myelome. Blood.

[23] Shaughnessy JD, Zhan F, Burington BE, Huang Y, Colla S, Hanamura I (2007). A validated gene expression model of high-risk multiple myeloma is defined by deregulated expression of genes mapping to chromosome 1. Blood.

[24] Garrido MM, Kelley AS, Paris J, Roza K, Meier DE, Morrison RS (2014). Methods for constructing and assessing propensity scores. Health Serv Res.

[25] Costa LJ, Abbas J, Hogan KR, Kramer C, McDonald K, Butcher CD (2012). Growth factor plus preemptive (‘just-in-time’) plerixafor successfully mobilizes hematopoietic stem cells in multiple myeloma patients despite prior lenalidomide exposure. Bone Marrow Transpl.

[26] Wehr VH, Corneau JM (2016). Efficacy and cost analysis of a plerixafor protocol for peripheral blood stem-cell mobilization in patients with multiple myeloma or non-hodgkin lymphoma. J Hematol Oncol Pharm.

[27] Micallef IN, Sinha S, Gastineau DA, Wolf R, Inwards DJ, Gertz MA (2013). Cost-effectiveness analysis of a risk-adapted algorithm of plerixafor use for autologous peripheral blood stem cell mobilization. Biol Blood Marrow Transpl.

[28] Douglas KW, Gilleece M, Hayden P, Hunter H, Johnson PRE, Kallmeyer C (2018). UK consensus statement on the use of plerixafor to facilitate autologous peripheral blood stem cell collection to support high-dose chemoradiotherapy for patients with malignancy. J Clin Apher.

[29] Duong HK, Savani BN, Copelan E, Devine S, Costa LJ, Wingard JR (2014). Peripheral blood progenitor cell mobilization for autologous and allogeneic hematopoietic cell transplantation: guidelines from the American Society for Blood and Marrow Transplantation. Biol Blood Marrow Transpl.

[30] Moreb JS, Byrne M, Shugarman I, Zou F, Xiong S, May WS (2018). Poor peripheral blood stem cell mobilization affects long-term outcomes in multiple myeloma patients undergoing autologous stem cell transplantation. J Clin Apher.

